# Report of a germline double heterozygote in *MSH2* and *PALB2*

**DOI:** 10.1002/mgg3.1242

**Published:** 2020-08-27

**Authors:** Konstantinos Agiannitopoulos, Eirini Papadopoulou, Georgios N. Tsaousis, Georgia Pepe, Stavroula Kampouri, Eleni Patsea, George Lypas, George Nasioulas

**Affiliations:** ^1^ Genekor Medical S.A Athens Greece; ^2^ Department of Genetic Oncology/Medical Oncology Hygeia Hospital Athens Greece; ^3^ Department of Pathology IASSO General Hospital Athens Greece

**Keywords:** endometrial cancer, *MSH2*, NGS, *PALB2*, pathogenic variant

## Abstract

**Background:**

Carriers with pathogenic variants in *MSH2* have increased risk to develop colorectal, endometrium, ovarian, and other types of cancer. The *PALB2* is associated with breast, ovarian, pancreatic, and prostate cancer. We describe the case of a 42‐year‐old female diagnosed with endometrial cancer at the age of 42 years with a strong family history of colorectal cancer, which was referred to our private diagnostic laboratory for genetic testing.

**Methods:**

In this study, we performed next‐generation sequencing (NGS) using an amplicon based 26 genes panel. The presence of multi‐exonic copy number variations (CNVs) was investigated by computational analysis and Multiplex Ligation‐dependent Probe Amplification (MLPA).

**Results:**

A gross deletion of the genomic region encompassing exons 11–16 of the *MSH2* and the loss‐of‐function variant c.757_758delCT, p.(Leu253Ilefs*3) in the *PALB2 *were identified in the proband.

**Conclusions:**

Multigene analysis using NGS technology allows the identification of pathogenic variants in genes that would normally not be tested based on the patient diagnosis. In our case these results explained not only the personal and/or family history of cancer but also allowed the surveillance for prevention of other cancer types. Moreover, the detection of large genomic rearrangements should be routinely included in hereditary cancer testing.

## Graphical abstract

We describe the case of a 42‐year‐old female diagnosed with endometrial cancer with a strong family history of colorectal cancer, which was referred to our private diagnostic laboratory for genetic testing. By next‐generation sequencing (NGS) and Multiplex Ligation‐dependent Probe Amplification (MLPA) analysis we identified a gross deletion of the genomic region encompassing exons 11–16 of the *MSH2* gene and the loss‐of‐function variant c.757_758delCT, p.(Leu253Ilefs*3) in the *PALB2* gene in our proband.

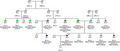

## 1 INTRODUCTION

The evolution of next‐generation sequencing (NGS) technologies has enabled for multigene panel analysis which is used in clinical practice for the identification of individuals with an inherited predisposition to cancer with the vast majority of them receiving results in genes for which clinical management guidelines are available (Slavin et al., 2015; Susswein et al., 2016).

Pathogenic variants in the *MSH2* (OMIM:609309) have been associated with Lynch syndrome (Hereditary Nonpolyposis Colorectal Cancer—HNPCC). *MSH2* mutation carriers have an increased risk of developing mainly colon and endometrial cancer. Moreover, pathogenic variants in *MSH2* have also been associated with ovarian, gastric, pancreatic, and brain tumors (Obermair et al., 2010).

Individuals who carry a pathogenic *PALB2* variant have an increased risk for autosomal dominant breast cancer, and possibly ovarian and pancreatic cancer (Rahman et al., 2007; Erkko et al., 2008; Antoniou et al., 2014). Additionally, the *PALB2* (OMIM:610355) is associated with autosomal recessive Fanconi anemia, type *N* (FA‐N). Patients carrying a pathogenic variant in the *PALB2* have a 14% risk of developing breast cancer by the age of 50 and a 35% risk by the age of 70 (Antoniou et al., 2014).

In this case study we report an individual who carries two pathogenic variants which are associated with a predisposition to *MSH2*‐ and *PALB2*‐related cancers. To our knowledge, clinical significant variants in these two different cancer genes have not been reported before simultaneously within one patient. Therefore, it is not known if these two pathogenic variants may interact and result in a difference in the cancer risks.

## 2 MATERIALS AND METHODS

### 2.1 Ethical compliance

Our study was approved by Hygeia hospital's scientific committee. The proband and the family members tested were informed about the significance of molecular testing, provided information about personal and family history and have signed an informed consent form prior to molecular genetic testing and permission for the anonymous use of their data for research purposes and/or scientific publications.

### 2.2 Patient

A 42‐year‐old female was diagnosed with endometrial cancer at the age of 42, and since colorectal, endometrium, ovarian, and kidney cancer was observed in her family, was referred to our private diagnostic laboratory for genetic testing with a hereditary cancer panel. Mismatch Repair (MMR) analysis by immunohistochemistry was carried out in the tumor tissue of the proband.

### 2.3 Gene testing

We collect peripheral blood samples for NGS analysis. Genomic DNA was extracted from peripheral blood leukocytes using MagCore^®^ Genomic DNA Whole Blood Kit (RBC Bioscience) according to the manufacturer's instructions.

The analysis of the entire coding region including the intron–exon boundaries of 26 genes involved in hereditary cancer predisposition was performed using the RUO BRCA Hereditary Cancer MASTR^™^ Plus assay kit (Multiplicom NV, Agilent) [*ABRAXAS1* (*FAM175A*) (NM_139076), *ATM* (*NM_000051*), *BLM* (NM_000057), *BARD1* (NM_000465), *BRCA1 *(NM_007294), *BRCA2 *(NM_000059), *BRIP1 *(NM_032043), *CDH1* (NM_004360), *CHEK2* (NM_007194), *EPCAM *(NM_002354), *MEN1* (NM_000244), *MLH1 *(NM_000249), *MRE11 *(*MRE11A*) (NM_005591), *MSH2 *(NM_000251), *MSH6* (NM_000179), *MUTYH *(NM_001128425), *NBN *(NM_002485), *PALB2* (NM_024675), *PMS2* (NM_000535), *PTEN* (NM_000314), *RAD50* (NM_005732), *RAD51C* (NM_058216), *RAD51D *(NM_002878), *STK11* (NM_000455), *TP53* (NM_000546), *XRCC2* (NM_005431)]. The sample preparation was performed according to the manufacturer's instructions. The sequencing was carried out using the Illumina MiSeq NGS technology and sequence changes were identified and interpreted in the context of a single clinically relevant transcript using the commercially available software suite SeqNext (JSI medical systems GmbH, Germany).

The presence of multi‐exonic copy number variations (CNVs), was investigated using the Multiplex Ligation‐dependent Probe Amplification (MLPA) method (MRC Holland) for the following genes: *BRCA1*, *BRCA2*, *CHEK2*, *EPCAM* (Exons 8, 9), *MLH1*, *MSH2*, *MSH6*, *MUTYH*, *PALB2*, *RAD50* (Exons 1, 2, 4, 10, 14, 21, 23 και 25), *RAD51C*, *RAD51D*, and *TP53*. MLPA analysis was performed using the appropriate MLPA probe mix and according to manufacturer's instructions: *BRCA1*: P002; *BRCA2*: P045; *CHEK2*: P190; *EPCAM*, *MSH6*: P072; *MLH1*, *MSH2*:P003; *MUTYH*: P378; *PALB2*, *RAD50*, *RAD51C*, *RAD51D*:P260; *TP53*:P056 (MRC Holland). Electrophoresis was achieved on an Applied Biosystems 3130 Genetic Analyzer (Applied Biosystems) and analysis was carried out using the Coffalyser.Net software. Moreover, the presence of multi‐exonic copy number variations (CNVs) was investigated using the commercial computational algorithm SeqPilot Version 4.4 Build 505 (JSI Medical System) using the NGS data.

Genomic DNA was extracted from peripheral blood leukocytes from siblings of the proband and was subjected to PCR using primers specific for the genetic location where the variant in the *PALB2* has been identified. Mutation analysis was carried out by Sanger sequencing and the results obtained were compared to reference sequences. Furthermore, MLPA for the *MSH2* multi‐exonic deletion analysis was performed using commercially available kit (SALSA MLPA P003 *MLH1*/*MSH2* probemix).

### 2.4 Variant classification and bioinformatics analysis

The clinical significance of variants was examined using the standards and guidelines for the interpretation of sequence variants recommended by the ACMG Laboratory Quality Assurance Committee and the Association for Molecular Pathology (AMP) (Richards et al., 2015). The impact of missense substitutions on protein structure and function was analyzed using computational predictive algorithms combined with the ensemble mutational impact score of MetaSVM (Dong et al., 2015). The effect on splicing was computationally examined using the Human Splicing Finder bioinformatics software (Desmet et al., 2009).

## 3 RESULTS

In our proband with endometrial cancer at the age of 42 years two pathogenic variants in the *MSH2* and *PALB2* were identified. The c.(1661+1_1662‐1‐(*1_?)del variant in *MSH2* was a gross deletion of the genomic region encompassing exons 11–16 and was detected using the SALSA MLPA P248 *MLH1*‐*MSH2* Confirmation probemix (Figure S1). This multi‐exonic deletion is expected to lead to the production of a truncated, inactive protein from one allele. This variant has been reported in individuals affected with Lynch syndrome (Martínez‐Bouzas et al., 2007; Romero et al., 2013) and has been described as pathogenic (https://www.ncbi.nlm.nih.gov/clinvar/variation/455060/). Additionally, the MMR analysis indicated significantly reduced expression of the MSH2 protein (5%), while the expression of MLH1, MSH6, and PMS2 proteins was 80%, 30%, and 60%, respectively (Figure S2).

The *PALB2* alteration c.757_758delCT,p.(Leu253Ilefs*3) was a deletion of two nucleotide bases in exon 4 of the gene, which led to a change of the reading frame and the generation of a termination codon after 3 amino acid residues (Figure S3). This variant has been identified in patients with breast and/or ovarian cancer (Casadei et al., 2011; Walsh et al., 2011; Caminsky et al., 2016; Shirts et al., 2016). Moreover, this variant has been reported to co‐occur with a second pathogenic *PALB2* variant in an individual affected with Fanconi anemia (Reid et al., 2007). It is also described as pathogenic by several laboratories in ClinVar (https://www.ncbi.nlm.nih.gov/clinvar/variation/126768/). No other pathogenic/likely pathogenic variants were identified in the remaining 24 genes that were analyzed.

**Figure 1:**
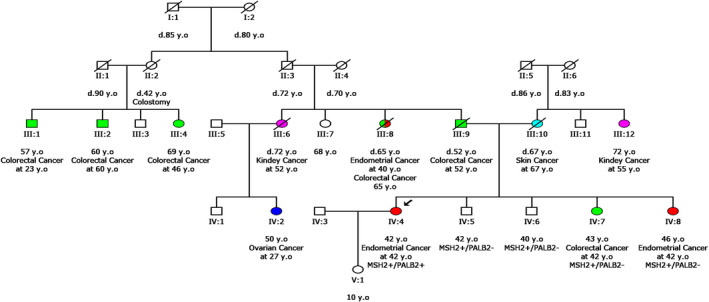
Pedigree of the proband's family. y.o, years old; d, died. Symbol +/− indicates test result. Different cancer types are represented with distinct colors

In the family history of the proband there were many cancer‐affected individuals, while colorectal and endometrial cancers were the prevalent tumor types (Figure 1). Additionally, all four siblings of this proband, two brothers (IV:5 and IV:6) and two sisters (IV:7 and IV:8) carried the *MSH2* but not the *PALB2* variant. The two sisters were affected by endometrial and colorectal cancer, respectively, phenotypes fully compatible with the *MSH2* alteration.

## 4 DISCUSSION

The patient's phenotype of endometrial cancer can be attributed to the presence of the *MSH2* variant. MMR results that show minimal expression of the MSH2 protein also support the inactivation of MSH2, indicating that most probably it could be the causative reason of tumor development. The identification of the *PALB2* pathogenic variant was an incidental finding, which, however, has a great impact not only for the family members of this individual but also for her surveillance and clinical management. Thus, this patient should follow surveillance guidelines for both the *MSH2*, which increases the risk mainly for colorectal and endometrial cancer (The National Comprehensive Cancer Network. Genetic/Familiar High‐Risk Assessment: Colorectal (Version 1. 2019, Accessed 3 July 2019), and for the *PALB2* mutation which confers increase risk for breast cancer (The National Comprehensive Cancer Network. Genetic/Familiar High‐Risk Assessment: Breast and Ovarian (Version 3. 2019, Accessed 18 January 2019). In addition, the identification of the *PALB2* pathogenic variant could also have future therapeutic implications for this patient since this gene is a member of the homologous recombination pathway, and its inactivation is known to confer impaired double strand break repair, a phenotype known as Homologous Recombination deficiency (HRD) (O'Kane, Connor, & Gallinger, 2017). HRD is associated with increased probability of response to targeted therapy with Poly(ADP‐ribose) polymerase inhibitors (PARPi) in multiple tumor types (Ohmoto & Yachida, 2017). Currently, germline and/or somatic mutations in HR genes are considered predictive markers of PARPi efficacy and are under investigation in several clinical trials. Preliminary results have shown that *PALB2* mutations could be predictive of PARPi treatment response in diverse tumor types (Mateo et al., 2019; Pilié et al., 2019; https://clinicaltrials.gov/). Thus, information on this gene could be useful in case of cancer recurrence, in the proband. Moreover, the proband's test results and the recommendation for testing close relatives (siblings), enabled their personalized clinical management and surveillance according to international guidelines.

It is expected that the detection of two pathogenic mutations in two different genes is associated with increased risk of developing cancers associated with each gene separately. In the international literature there are no reports of patients carrying mutations in these two genes at the same time. As a result, it is not possible to predict whether the effect of these two variants interacts, thus changing the phenotype of the subject. The main limitation of this study was the absence of genetic material from the proband's mother and father who were diagnosed with skin and colorectal cancer, respectively.

In the past whenever a patient with cancer family history was referred for genetic analysis, it was common clinical practice to perform sequential analysis of the one or the few genes that suited more to his phenotype. However, single gene analysis apart from being laborious and time consuming, it has also the disadvantage of leading to the termination of the analysis whenever a positive finding was identified. By using NGS technology, it has become possible to study a wider range of hereditary cancer‐related genes simultaneously. This has an important clinical impact since, as observed in previous studies (Crawford et al., 2017; LaDuca et al., 2014; Tsaousis et al., 2019) a considerable percentage of individuals with pathogenic findings, present clinical significant variants in more than one gene. Each altered gene can independently increase the risk of different tumor types, while their combined effect in many cases has not been studied. For example, an individual carrying mutation in a breast cancer risk gene and in a gene conferring increased risk of colorectal cancer should take in consideration these findings and follow surveillance guidelines for both alterations. In addition, the patient's relatives should consider testing for both alterations detected. If the cancer phenotype had been attributed only to the first alteration identified some of the carrier's relatives negative for the first alteration and carrying the second alteration would have been misclassified as of low risk for cancer development.

In conclusion, NGS technology enables the analysis of multigene panels and can accelerate the identification of the causative etiology of cancer phenotype in the proband's family. Furthermore, this analysis can be more informative compared to single gene analysis and lead to the identification of multiple clinical findings that have an impact both on the clinical management of the individual examined and on the more accurate determination of the cancer risk in his relatives.

## CONFLICT OF INTEREST

The authors state no conflict of interest.

## AUTHOR CONTRIBUTIONS

KA, EP, and GNT drafted the manuscript. KA and EP designed the study and the sequencing experiments, and coordinated the study. KA, GP, and SK carried out the DNA extraction, sequencing and contributed to the analysis and interpretation of the variant data. GNT performed the bioinformatics analysis. EP performed the Mismatch Repair (MMR) analysis by immunohistochemistry. GL provided the proband material, demographic data, family history, and management. GN conceived of the study, and participated in its design and coordination. All authors read and approved the final manuscript.

## Supporting information

Fig S1‐S3
