# Supplementary material for: Report of a germline double heterozygote in MSH2 and PALB2
Source: Mol Genet Genomic Med. 2020 Aug 27;8(10):e1242. doi: 10.1002/mgg3.1242 (PMC7549547; doi:10.1002/mgg3.1242)
Supplement: Supplementary file 1 — Fig S1‐S3 [file MGG3-8-e1242-s001.pdf]

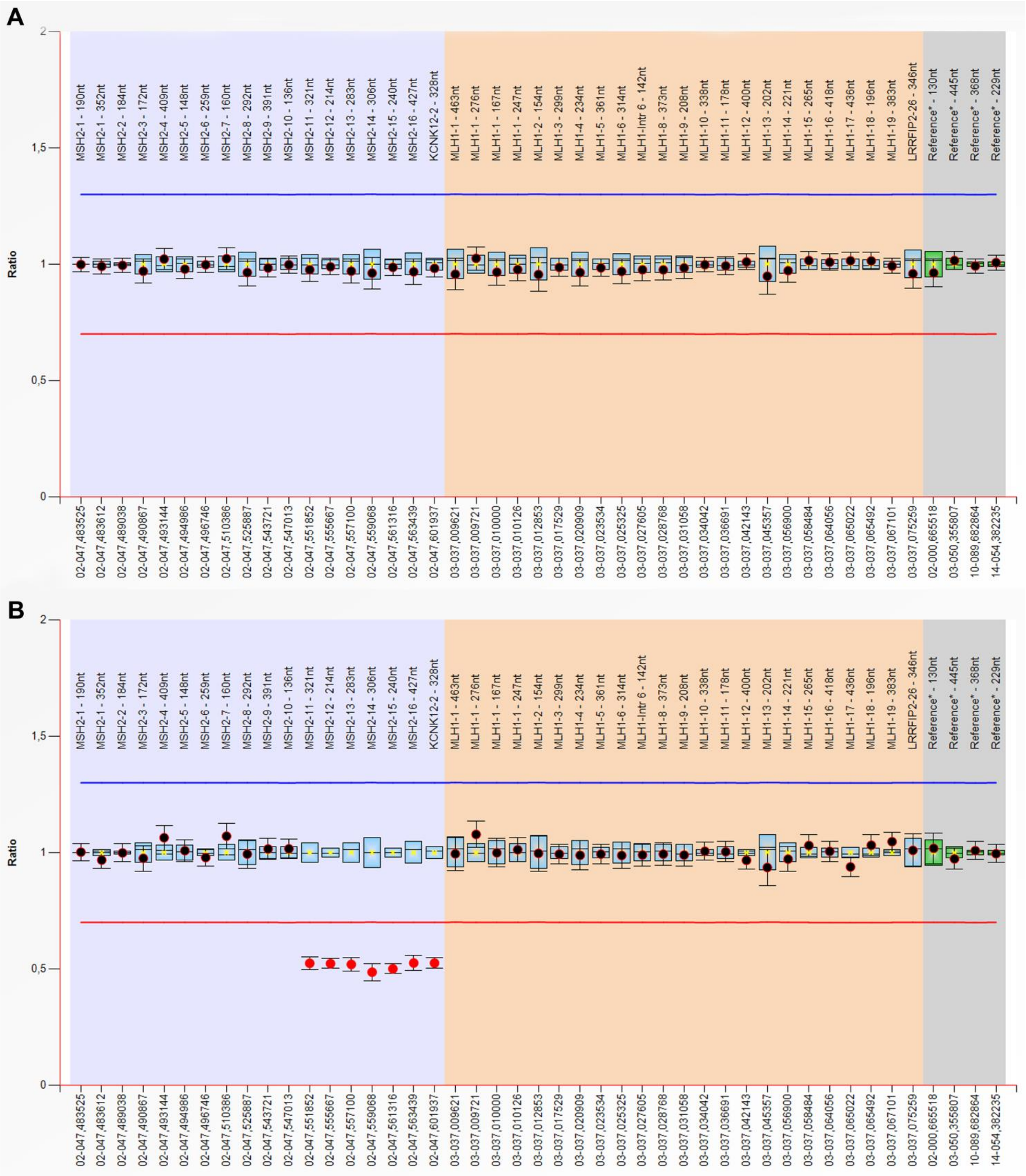

Figure S1. Representative plots of the MLPA analysis by Coffalyser.Net showing the probe ratios with 95% confidence intervals as error bars for all exons of the *MSH2* gene. **A.** MLPA analysis of the normal reference sample; **B.** MLPA analysis of the proband showing heterozygous deletion of exons 11-16 in the *MSH2* gene.

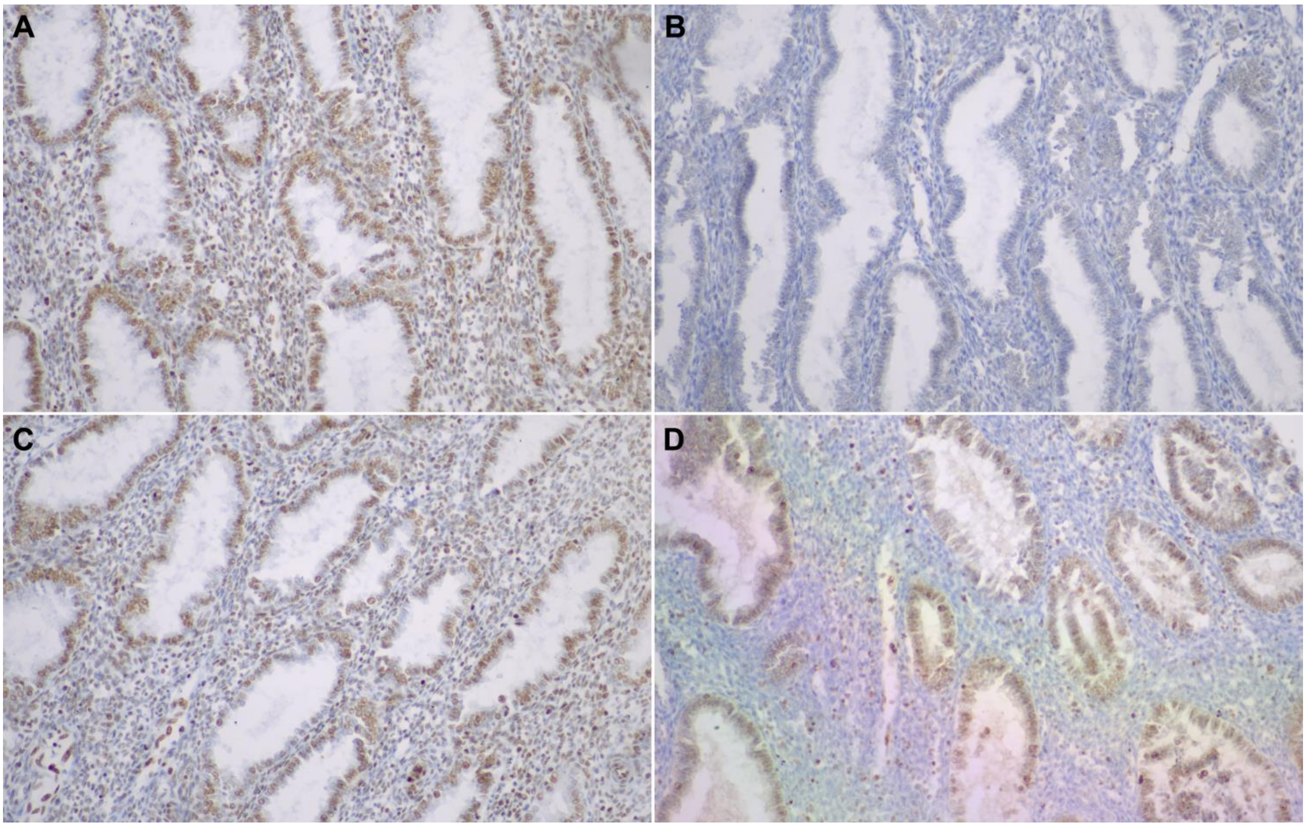

Figure S2. Immunohistochemical analysis of the expression of all four MMR proteins**: A**. MLH1 (antibody M1, ROCHE); **B.** MSH2 (antibody G219-1129, ROCHE); **C.** MSH6 (SP93, ROCHE) and **D.** PMS2 (antibody A16-4, ROCHE).

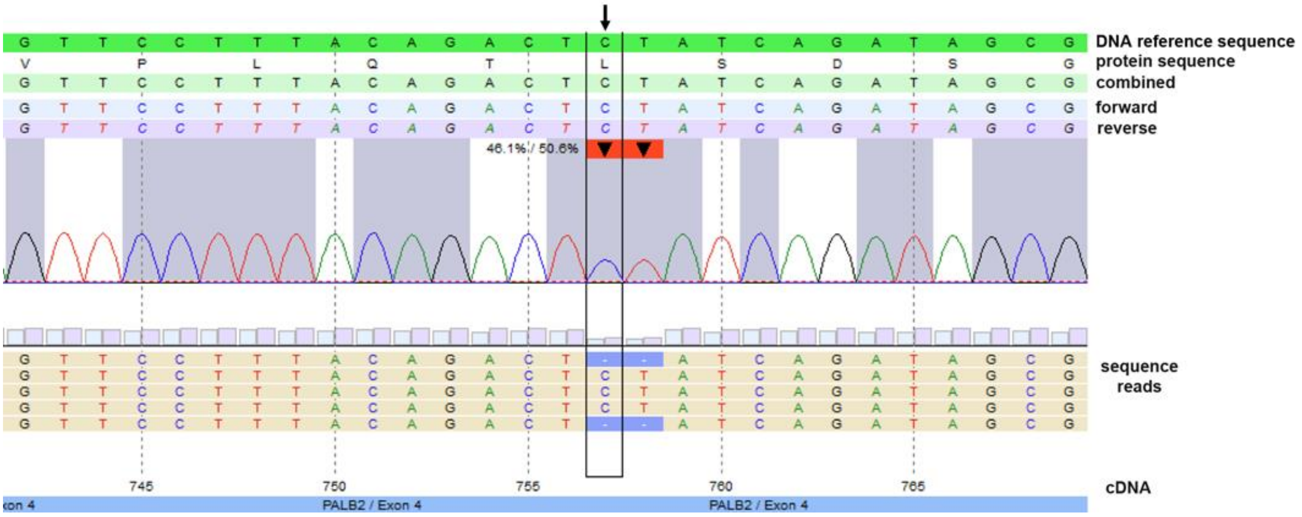

Figure S3. Next generation DNA sequencing pseudo-electropherogram demonstrating heterozygosity for the c.757_758delCT, p.(Leu253Ilefs*3) variant in the *PALB2* gene in our patient. Results of forward and reverse DNA sequencing of exon 4 analyzed by SeqNext. The mutation is indicated with a rectangle indicated and an arrow.
